# Supplementary figures and images for: Surface colonization by Flavobacterium johnsoniae promotes its survival in a model microbial community
Source: mBio. 2024 Feb 8;15(3):e03428-23. doi: 10.1128/mbio.03428-23 (PMC10936215; doi:10.1128/mbio.03428-23)

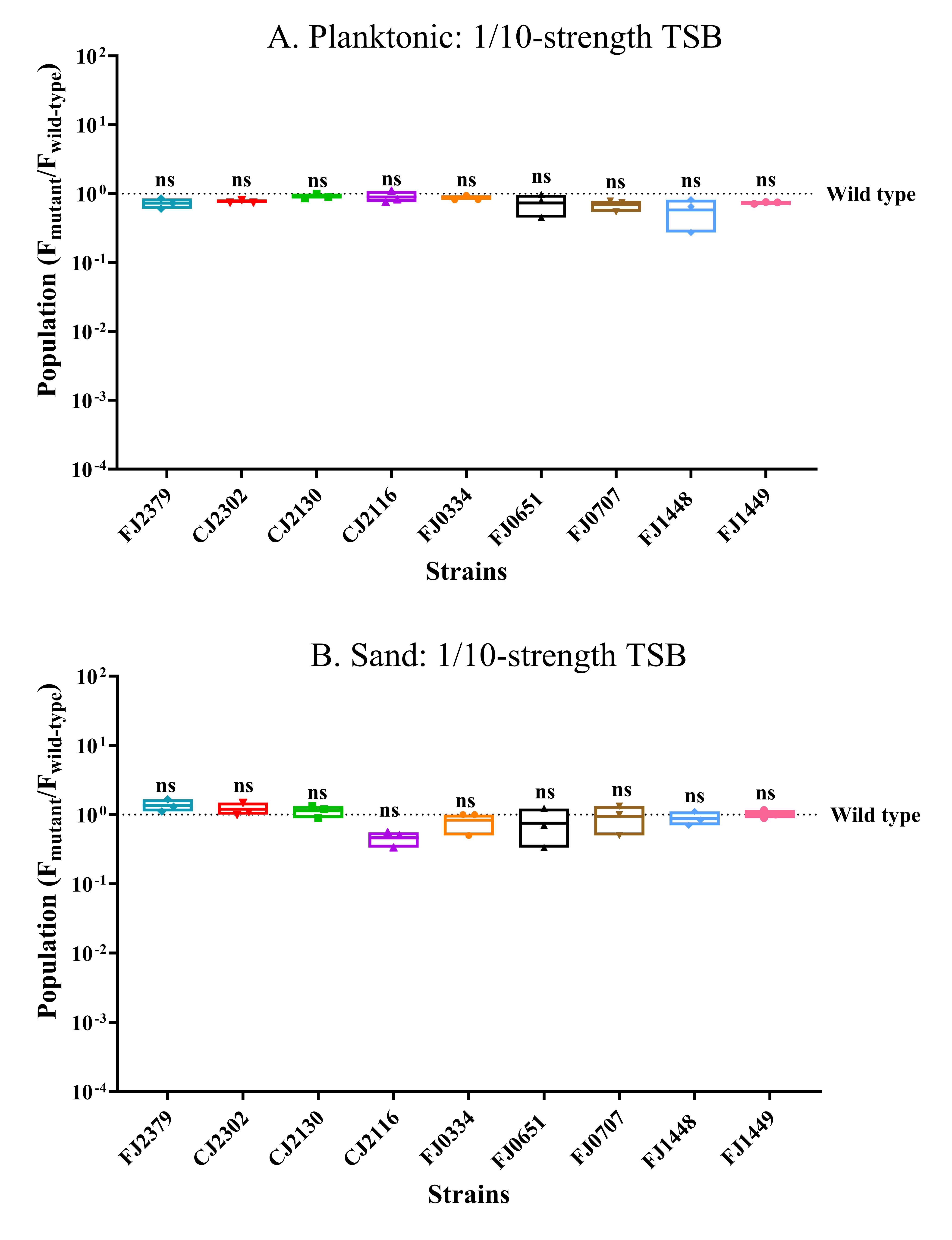

Supplement: Figure S1 — Sand colonization by deletion mutants complemented with genes of interest. [file mbio.03428-23-s0001.tif]

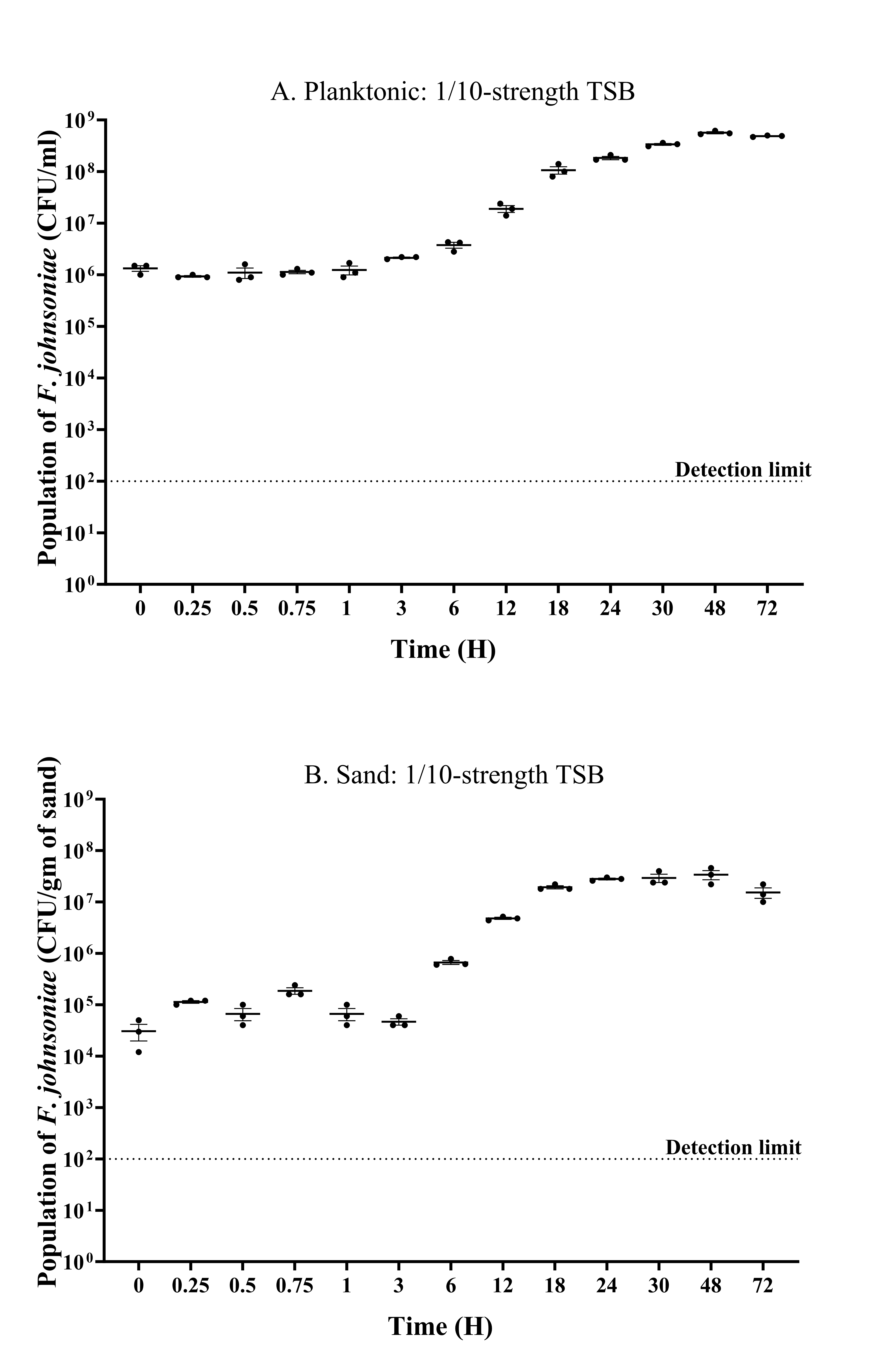

Supplement: Figure S2 — Sand colonization by F. johnsoniae CJ1827 (wild type) over time. [file mbio.03428-23-s0002.tif]

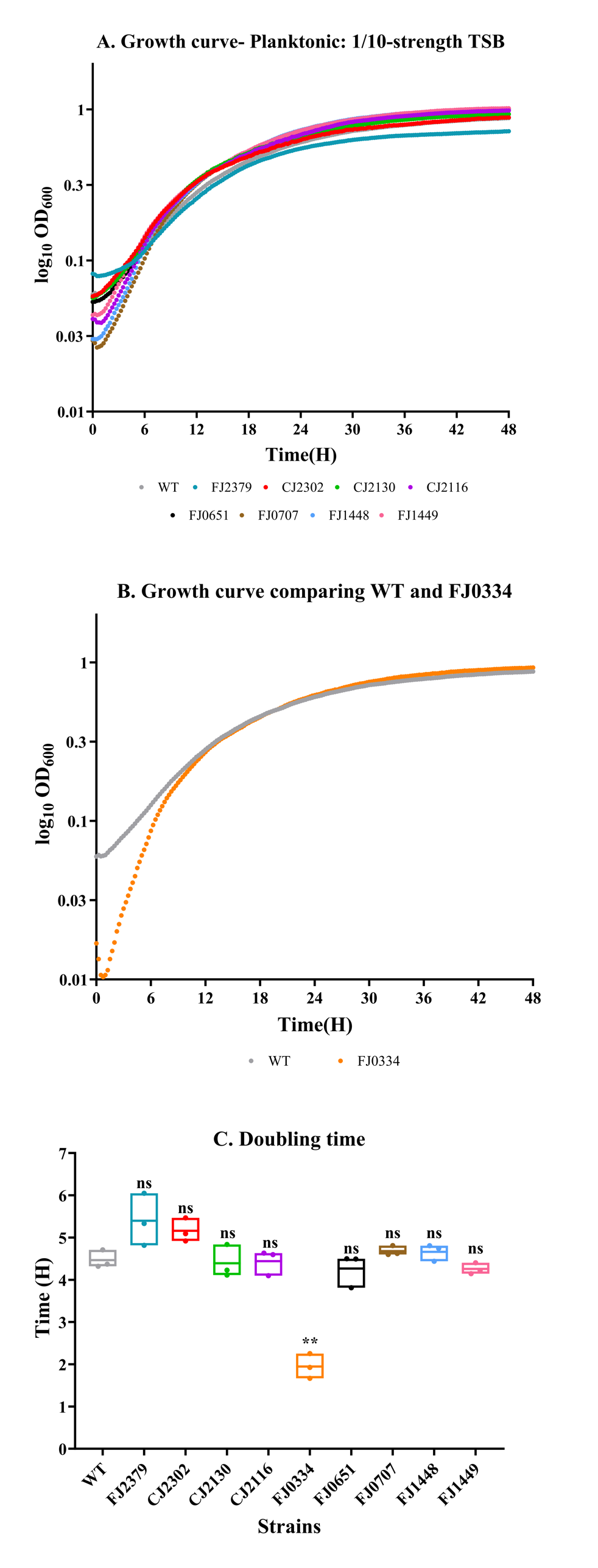

Supplement: Figure S3 — Growth kinetics of wild type and mutants in 1/10-strength TSB. [file mbio.03428-23-s0003.tif]

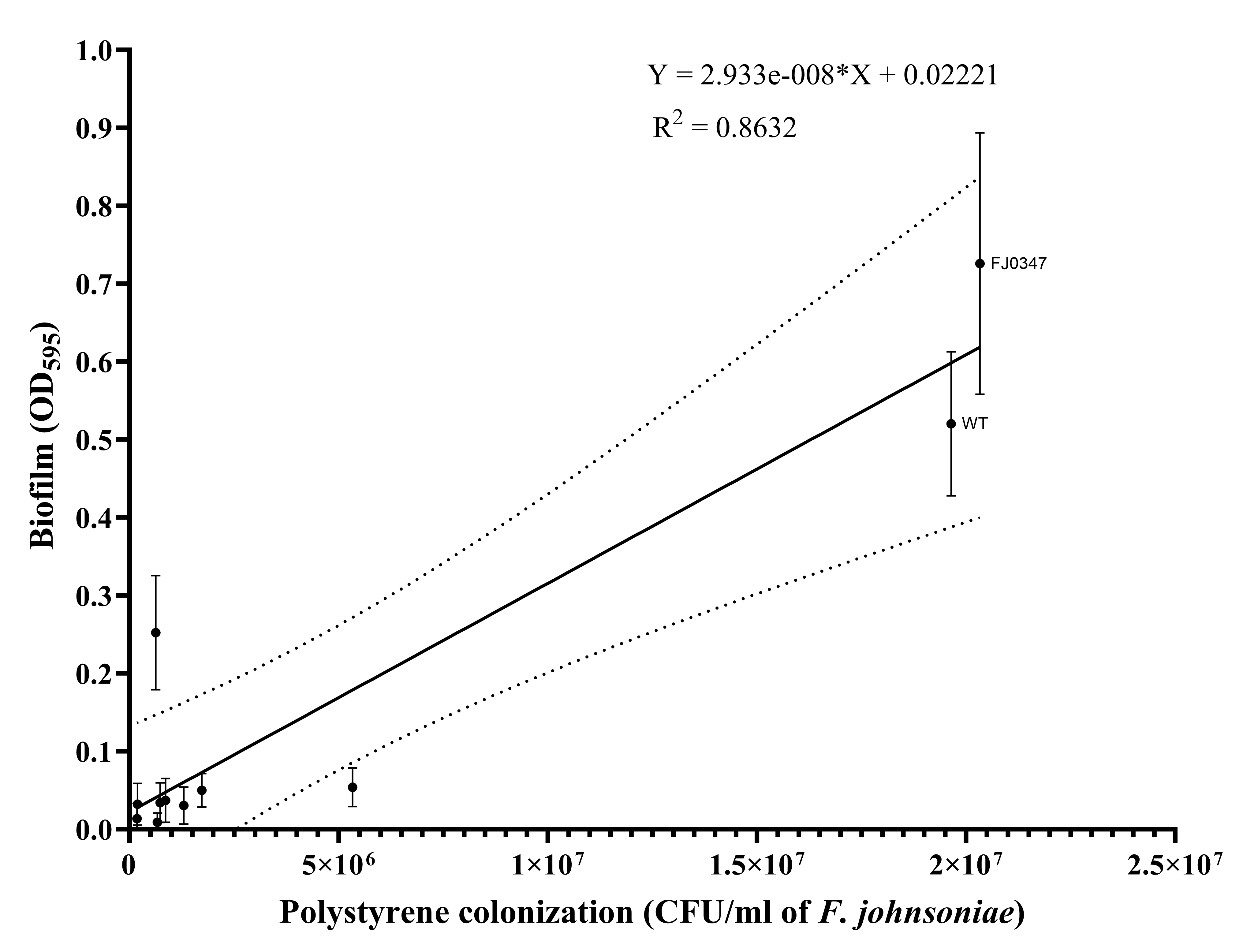

Supplement: Figure S5 — Linear regression analysis of the relationship between polystyrene colonization and biofilm formation. [file mbio.03428-23-s0005.tif]

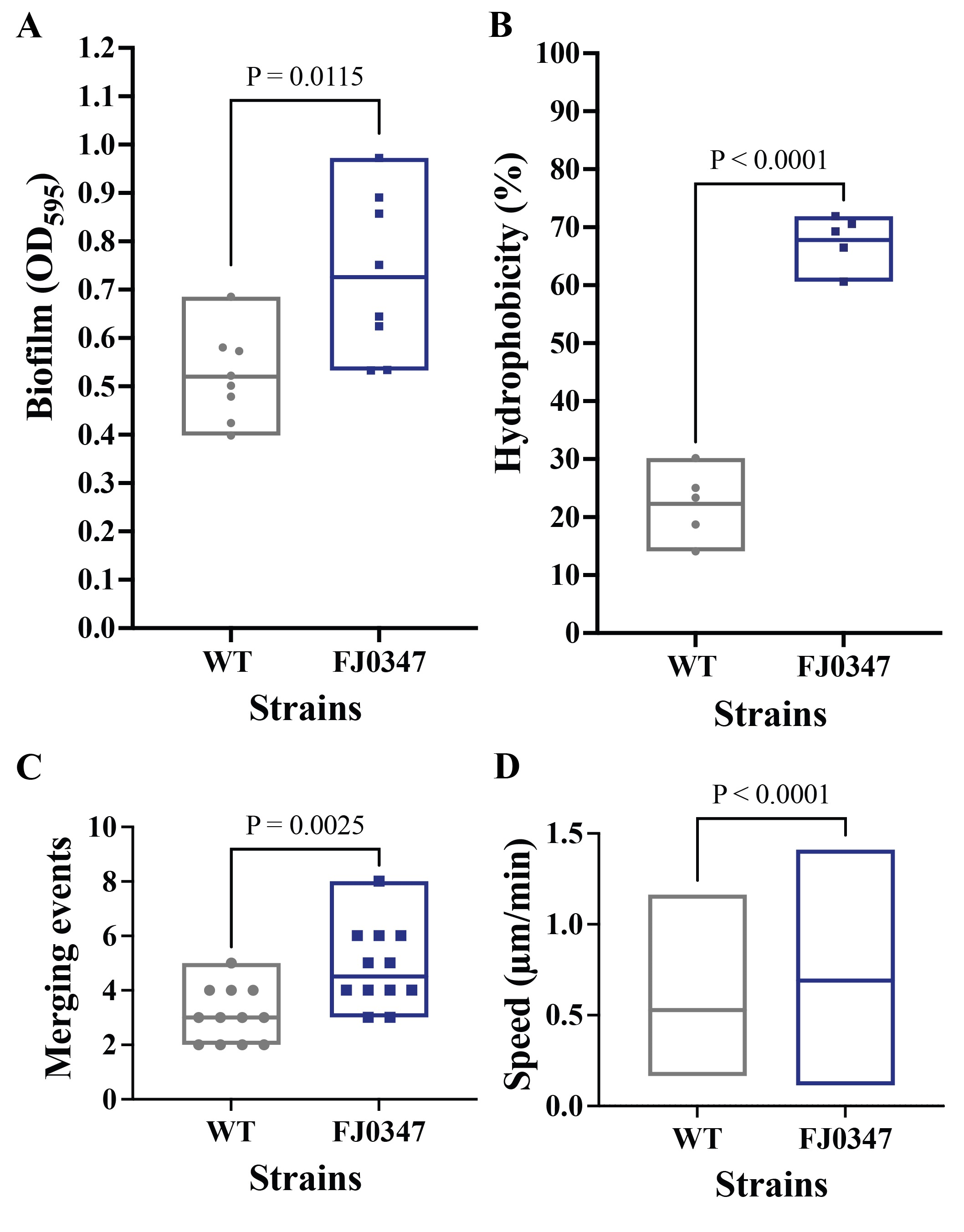

Supplement: Figure S6 — Characterization of overrepresented mutant FJ0347. [file mbio.03428-23-s0006.tif]
