# Supplementary material for: Surface colonization by Flavobacterium johnsoniae promotes its survival in a model microbial community
Source: mBio. 2024 Feb 8;15(3):e03428-23. doi: 10.1128/mbio.03428-23 (PMC10936215; doi:10.1128/mbio.03428-23)
Supplement: Supplemental text — Captions for supplemental figures and movie. [file mbio.03428-23-s0007.docx]

**SUPPLEMENTARY MATERIAL**

**Fig. S1: Sand colonization by deletion mutants complemented with genes of interest**. Populations of wild type *F. johnsoniae* CJ1827 and complemented strains are represented as ratio of complemented strain to the wild type. The initial inoculum was 10^6^ CFU/ml of wild type *F. johnsoniae* CJ1827 or complemented strains. Populations were determined after 48 h under the following conditions: (A) no solid substrate (planktonic: 1/10-strength TSB), (B) sand: 1/10-strength TSB. The dotted line indicates the wild type and each data point in box plots represents one biological replicate. Deletion mutants complemented with underrepresented genes are in red and the deletion mutant complemented with the overrepresented gene is in blue. Statistical significance was evaluated using One-way ANOVA followed by Dunnett’s test. Differences between mutant and the wild type are indicated as ns, not significant.

**Fig. S2: Sand colonization by *F. johnsoniae* CJ1827 (wild type) over time.** The population of *F. johnsoniae* was determined at various time points in two conditions: (A) no solid substrate (planktonic: 1/10-strength TSB), (B) sand: 1/10-strength TSB. The dotted line indicates the limit of detection (10^2^ CFU/mL) and each data point in box plots represents one biological replicate.

**F****ig. S3: Growth kinetics of wild-type and mutants in 1/10-strength TSB.** The absorbance (OD_600_) of wild type and mutants were measured at various time points for 48 h. (A) Growth curve of wild type *F. johnsoniae* CJ1827 (WT) and mutants (FJ2379, CJ2302, CJ2130, CJ2116, FJ0651, FJ0707, FJ1448, FJ1449). (B) Growth curve of wild type *F. johnsoniae* CJ1827 (WT) and FJ0334. Each dot in the growth curve represents the mean of 3 biological and 3 technical replicates of wild type and mutants. (C) Doubling time of *F. johnsoniae* strains calculated during exponential phase. Statistical significance was evaluated using One-way ANOVA followed by Dunnett’s test. Differences between mutant and the wild type are indicated as ns, not significant, **, p < 0.01.

**Fig. S4: Colony spreading of wild type and colonization defective mutants in 1% PY2 agar.** To observe colony spreading, *F. johnsoniae* strains were grown overnight in CYE medium at 28 °C with shaking. The cells were pelleted, washed, and resuspended in 1X PBS and 10 mL was spotted onto peptone yeast (PY2) agar. The plates were incubated in 28^o^C and photographed after 5 days. The experiment was conducted three times, and representative results are presented.

**Fig. S5: Linear regression analysis of the relationship between polystyrene colonization and biofilm formation.** The graph displays data from both the wild type and colonization mutant; 99% confidence intervals represent the best-fit line, slope, and R^2^ value.

**Fig. S6:** **Characterization of overrepresented mutant FJ0347.** (A) Biofilm formed by overrepresented mutant, FJ0347, and the wild type (WT). Each point represents one biological replicate with data averaged from eight technical replicates. (B) Bacterial cell-surface hydrophobicity of FJ0347 and WT. The hydrophobicity is represented as [1-(A_final_/A_intial_)]*100, where A_final_ is the absorbance of the aqueous phase after phase separation, and A_intial_ is the initial absorbance of the strain. (C) Number of merging events throughout the lifetime of a given zorb for wild type and FJ0347. Each data point in the plot represents an individual zorb tracked between 8 and 16 h. (D) Mean zorb speed for the wild type and FJ0347. Statistical significance was determined by an independent two-sample t-test.

**Movie S1: Bacterial zorbing time-lapse videos.** Bright-field time-lapse videos of zorb formation and dispersion of wild type (CJ1827) and representative mutants (FJ0347, FJ2379, FJ0334) over a period of 16 h.
