# Supplementary material for: Surface colonization by Flavobacterium johnsoniae promotes its survival in a model microbial community
Source: mBio. 2024 Feb 8;15(3):e03428-23. doi: 10.1128/mbio.03428-23 (PMC10936215; doi:10.1128/mbio.03428-23)
Supplement: Table S1 — Complete list of sand colonization genes identified using INSeq. [file mbio.03428-23-s0008.docx]

**Table S1: Complete list of sand colonization genes identified using INSeq.** The COG categories are abbreviated as follows: E-amino acid metabolism and transport; T-signal transduction; S-unknown function; I-lipid transport and metabolism; M-cell wall/membrane/envelop biogenesis; Q-secondary metabolite biosynthesis; F-Nucleotide transport and metabolism; O-Post-translational modification; P-Inorganic ion transport and metabolism; C-Energy production and conversion.

| Accession ID | Gene | Annotation | COG Category | Log2 Fold change (Input/Output) |
| --- | --- | --- | --- | --- |
| Underrepresented genes | | | | |
| Fjoh_0295 | *FJOH_RS01575* | glycosyltransferase family 2 protein | S | 1.19 |
| Fjoh_0334 | *FJOH_RS01770* | DegT/DnrJ/EryC1/StrS family aminotransferase | E | 2.54 |
| Fjoh_0361 | *FJOH_RS01905* | polysaccharide biosynthesis/export family protein | M | 1.45 |
| Fjoh_0402 | *FJOH_RS02105* | membrane protein | M | 1.51 |
| Fjoh_0450 | *FJOH_RS02345* | ATP-binding cassette domain-containing protein | Q | 3.01 |
| Fjoh_0539 | *FJOH_RS02845* | hypothetical protein | S | 1.4 |
| Fjoh_0627 | *FJOH_RS03290* | hypothetical protein | no COG | 1.21 |
| Fjoh_0651 | *FJOH_RS03415* | hypothetical protein | no COG | 2.14 |
| Fjoh_0707 | *FJOH_RS03710 (porZ)* | T9SS type A sorting domain-containing protein (porZ) | T | 1.48 |
| Fjoh_1028 | *FJOH_RS05365* | hypothetical protein | T | 1.03 |
| Fjoh_1448 | *xrtF* | exosortase family protein XrtF | S | 3.82 |
| Fjoh_1449 | *FJOH_RS07530* | exosortase F system-associated protein | no COG | 3.74 |
| Fjoh_1555 | *porV* | type IX secretion system outer membrane channel protein PorV | I | 2.69 |
| Fjoh_1556 | *porU* | type IX secretion system sortase PorU | S | 1.33 |
| Fjoh_1653 | *sprA* | cell surface protein SprA | S | 3.25 |
| Fjoh_1656 | *deoC* | deoxyribose-phosphate aldolase | F | 1.37 |
| Fjoh_2379 | *FJOH_RS12370* | LysM peptidoglycan-binding domain-containing protein | M | 2.5 |
| Fjoh_2557 | *FJOH_RS13270* | cysteine synthase family protein | E | 2.85 |
| Fjoh_3499 | *FJOH_RS18100* | DoxX family protein | O | 2.96 |
| Fjoh_4507 | *FJOH_RS23285* | chloride channel protein | P | 2.1 |
| Fjoh_4988 | *FJOH_RS25770* | 2-oxo acid dehydrogenase subunit E2 | C | 1.71 |
| Overrepresented genes | | | | |
| Fjoh_0347 | *FJOH_RS01835* | nucleotidyltransferase | M | -1.11 |
| Fjoh_2046 | *FJOH_RS10655* | L-rhamnose mutarotase | S | -5.03 |
| Fjoh_2837 | *FJOH_RS14730* | nucleoside phosphorylase | F | -1.63 |
| Fjoh_3043 | *FJOH_RS15790* | hypothetical protein | no COG | -2.05 |
