# Supplementary material for: Surface colonization by Flavobacterium johnsoniae promotes its survival in a model microbial community
Source: mBio. 2024 Feb 8;15(3):e03428-23. doi: 10.1128/mbio.03428-23 (PMC10936215; doi:10.1128/mbio.03428-23)
Supplement: Table S2 — Primers used in this study. [file mbio.03428-23-s0009.docx]

**Table S2: List of primers used in this study.**

| **Primers** | **Sequence** |
| --- | --- |
| **Fjoh_0334_UF** | 5’CACC**GGATCC**CGCTTACGTCGTAGCGATTG 3’; used in  construction of FJ0334; BamHI site is in bold |
| **Fjoh_0334_UR** | 5’AGTACTCTATTCTTTGTCCTAATTATCcatTTATGAGCCAATAATCTGAAGGAT 3’; used in construction of FJ0334 |
| **Fjoh_0334_DF** | 5’TGGCTCATAAatgGATAATTAGGACAAAGAATAGAGTACTATATTTAAAATGAAAATTGAAAAAAC 3’; used in construction of FJ0334 |
| **Fjoh_0334_DR** | 5’**GGATCC**AGAGCAGAAGATGAAGACCCATC 3’; used in  construction of FJ0334; BamHI site is in bold |
| **Fjoh_0334_SEAM** | 5’CGCATGGCAGGGACACA 3’; used to confirm deletion of *fjoh_0334* |
| **Fjoh_0347_UF** | 5’CACC**GGATCC**GGTTCTTCAGAAATATTTCATAAGGCTGG 3’; used in construction of FJ0347; BamHI site is in bold |
| **Fjoh_0347_UR** | 5’AGAACTTTGTATTTtcaTCTAATTaatCTTGCTCATATTTATCATTATTTGCGC 3’; used in construction of FJ0347 |
| **Fjoh_0347_DF** | 5’AAATATGAGCAAGattAATTAGAtgaAAATACAAAGTTCTGATTTTTCTATCATCATAC 3’; used in construction of FJ0347 |
| **Fjoh_0347_DR** | 5’**GGATCC**CGACAGCAATACTTGCCTCTTTTC 3’; used in  construction of FJ0347; BamHI site is in bold |
| **Fjoh_0347_SEAM** | 5’AAGGATGGTTTATTGGAAATTTCGAACC 3’; used to confirm deletion of *fjoh_0347* |
| **Fjoh_0651_UF** | 5’CACC**GGATCC**GGTGTTCAAAACTGCGCTGT  GGCAAAAACTAAATTGAAGTACAATTCG 3’; used in  construction of FJ0651; BamHI site is in bold |
| **Fjoh_0651_UR** | 5’ATAAAAAAAGCTTCAATAAAATTTGGGGGTGCTG 3’; used in  construction of FJ0651 |
| **Fjoh_0651_DF** | 5’ATCAGCACCCCCAAATTTTATTGAAGCTTTTTTTATGAAGTAAATTTTCTTTCTTATTTG 3’; used in construction of FJ0651 |
| **Fjoh_0651_DR** | 5**’GGATCC**TTCTTTTAAAGCCTTTGTAGCCGC 3’; used in  construction of FJ0651; BamHI site is in bold |
| **Fjoh_0651_SEAM** | 5’ATAATCAATACTATTATA 3’; used to confirm deletion of *fjoh_0651* |
| **Fjoh_0707_UF** | 5’CACC**GGATCC**GATCTTGATGTTCCTGCCGGA 3’; used in  construction of FJ0707; BamHI site is in bold |
| **Fjoh_0707_UR** | 5’ATTATATAAAACTGAAAATATTTGATATTTTCAGATAGACAAATATACTACAAACGAC 3’; used in construction of FJ0707 |
| **Fjoh_0707_DF** | 5’ATCTGAAAATATCAAATATTTTCAGTTTTATATAATCTACAATCTACAATATAAAATTGTGC 3’; used in construction of FJ0707 |
| **Fjoh_0707_DR** | 5’**GGATCC**CCTTGGCATCTTTAAACAAGGCA 3’; used in  construction of FJ0707; BamHI site is in bold |
| **Fjoh_0707_SEAM** | 5’CTATGTAAAAGGAGCCAACATTGCC 3’; used to confirm deletion of *fjoh_0707* |
| **Fjoh_1448_UF** | 5’CACC**GGATCC**CGCTTCCTTCCCAAGAACCT 3’; used in  construction of FJ1448; BamHI site is in bold |
| **Fjoh_1448_UR** | 5’TACTATTctaTTTAGCATCAGATTTTTTTTTCAAATTTATGTATTTTTTGAGTTTAGCT 3’; used in construction of FJ1448 |
| **Fjoh_1448_DF** | 5’ATTTGAAAAAAAAATCTGATGCTAAAtagAATAGTAGAGCAAAAGAAGAAAATTTTTATC 3’; used in construction of FJ1448 |
| **Fjoh_1448_DR** | 5’**GGATCC**CGAAAGCAAAAACACCACAAAAAGC 3’; used in  construction of FJ1448; BamHI site is in bold |
| **Fjoh_1448_SEAM** | 5’GGCACCCACATAAGGGCC 3’; used to confirm deletion of *fjoh_1448* |
| **Fjoh_1449_UF** | 5’CACC**GGATCC**TCGGCTTCTGTAAAAGGGTCG 3’; used in  construction of FJ1449; BamHI site is in bold |
| **Fjoh_1449_UR** | 5’TAAAAATCTAATAAAATGCTATCTATTTAGcatATTTTGAAAATTTATTAACCCATAAAACCC 3’; used in construction of FJ1449 |
| **Fjoh_1449_DF** | 5’TCAAAATatgCTAAATAGATAGCATTTTATTAGATTTTTATTCAAACCATTTTAGGTATTTTTGC 3’; used in construction of FJ1449 |
| **Fjoh_1449_DR** | 5’**GGATCC**AAGCTCGGTATTTATTTGTCCCGAAA 3’; used in  construction of FJ1449; BamHI site is in bold |
| **Fjoh_1449_SEAM** | 5’AGTTACGCTGCTTTATATTTTGTTCGG 3’; used to confirm deletion of *fjoh_1449* |
| **Fjoh_2379_UF** | 5’CACC**GGATCC**CTCGGTAGAAAAACTGCGTTTGG 3’; used in  construction of FJ2379; BamHI site is in bold |
| **Fjoh_2379_UR** | 5’AAGTTACTTTTGATGTCAAATAAAGCTTAAAACAATTTTAATTTCTAATTCAAAAACAAGATAC 3’; used in construction of FJ2379 |
| **Fjoh_2379_DF** | 5’ATTGTTTTAAGCTTTATTTGACATCAAAAGTAACTTACTTAGGCGA 3’; used in construction of FJ2379 |
| **Fjoh_2379_DR** | 5’**GGATCC**CCTTCTTCTTTATGCAGACGCAC 3’; used in  construction of FJ2379; BamHI site is in bold |
| **Fjoh_2379_SEAM** | 5’CAGTGCAAGAAAAAGGAAGAAGATGAG 3’; used to confirm deletion of *fjoh_2379* |
| **Fjoh_0334_CP_UP** | 5’GGTGGT**GGATCC**atgGATAATTCAAGAATTTATTTATCACTCTCAG 3’; used in the construction of pRR0334; BamHI site is in bold |
| **Fjoh_0334_CP_DOWN** | 5’GGTGGT**GCATGC**tcaATTAAAAAATTCTTCAATTACTTTTCTAATTCTATCC 3’; used in the construction of pRR0334; SphI site is in bold |
| **Fjoh_0347_CP_UP** | 5’GGTGGT**GGATCC**ATGAGCAAGATTAATATAGTAATCCCTATGGC 3’; used in the construction of pRR0347; BamHI site is in bold |
| **Fjoh_0347_CP_DOWN** | 5’GGTGGT**GCATGC**TCATAATCTCTTTAAATATACTTCTAAATCTTCAGGTGTTC 3’; used in the construction of pRR0347; SphI site is in bold |
| **Fjoh_0651_CP_UP** | 5’GGTGGT**GGATCC**GGCAAAAACTAAATTGAAGTACAATTCG 3; used in the construction of pRR0651; BamHI site is in bold |
| **Fjoh_0651_CP_DOWN** | 5’GGTGGT**GCATGC**ttaAATTGAAGCTTTTTTTATTTTATTTCGATATATAACTG 3’; used in the construction of pRR0651; SphI site is in bold |
| **Fjoh_0707_CP_UP** | 5’GGTGGT**GGATCC**atgGTAAAAAGATTTCTGTGCTTTTTGT 3’; used in the construction of pRR0707; BamHI site is in bold |
| **Fjoh_0707_CP_DOWN** | 5’GGTGGT**GCATGC**ttaTCGAATAATCATAACCTTCTTAACCTTTG 3’; used in the construction of pRR0707; SphI site is in bold |
| **Fjoh_1448_CP_UP** | 5’GGTGGT**GGATCC**GCACTACAAAATTTGCATTGCTTTC 3’; used in the construction of pRR1448; BamHI site is in bold |
| **Fjoh_1448_CP_DOWN** | 5’GGTGGT**GCATGC**ctaTTTAGCATATTTTGAAAATTTATTAACCCATAAAAC 3’; used in the construction of pRR1448; SphI site is in bold |
| **Fjoh_1449_CP_UP** | 5’GGTGGT**GGATCC**atgCTAAATAGAATAGTAGAGCAAAAGAAGA3’; used in the construction of pRR1449; BamHI site is in bold |
| **Fjoh_1449_CP_DOWN** | 5’GGTGGT**GCATGC**ttaTTTTTTTAAATTTTGAAATTGATAATAAAATGCAGGAA 3’; used in the construction of pRR1449; SphI site is in bold |
| **Fjoh_2379_CP_UP** | 5’GGTGGT**GGATCC**GGTATATTTACAGACGCAGTACCAAG 3’; used in the construction of pRR2379; BamHI site is in bold |
| **Fjoh_2379_CP_DOWN** | 5’GGTGGT**GCATGC**ttaATTTGCTGCTTTTAAAGTTAAGTCTGA 3’; used in the construction of pRR2379; SphI site is in bold |
